# Language and music phrase boundary processing in Autism Spectrum Disorder: An ERP study

**DOI:** 10.1038/s41598-017-14538-y

**Published:** 2017-10-31

**Authors:** John DePriest, Anastasia Glushko, Karsten Steinhauer, Stefan Koelsch

**Affiliations:** 1 Freie Universität Berlin, Berlin, Germany; 2 Program in Linguistics, Tulane University, New Orleans, Louisiana United States of America; 3 School of Communication Sciences and Disorders, McGill University, Montreal, Quebec Canada; 4 The Centre for Research on Brain, Language and Music (CRBLM), Montreal, Quebec Canada; 5 University of Bergen, Bergen, Norway

## Abstract

Autism spectrum disorder (ASD) is frequently associated with communicative impairment, regardless of intelligence level or mental age. Impairment of prosodic processing in particular is a common feature of ASD. Despite extensive overlap in neural resources involved in prosody and music processing, music perception seems to be spared in this population. The present study is the first to investigate prosodic phrasing in ASD in both language and music, combining event-related brain potential (ERP) and behavioral methods. We tested phrase boundary processing in language and music in neuro-typical adults and high-functioning individuals with ASD. We targeted an ERP response associated with phrase boundary processing in both language and music – i.e., the Closure Positive Shift (CPS). While a language-CPS was observed in the neuro-typical group, for ASD participants a smaller response failed to reach statistical significance. In music, we found a boundary-onset music-CPS for both groups during pauses between musical phrases. Our results support the view of preserved processing of musical cues in ASD individuals, with a corresponding prosodic impairment. This suggests that, despite the existence of a domain-general processing mechanism (the CPS), key differences in the integration of features of language and music may lead to the prosodic impairment in ASD.

## Introduction

A large subgroup of individuals with autism spectrum disorder (ASD) shows deficient language abilities^1^. One of the most prominent difficulties within the language domain is the impairment in prosodic processing^2,3^, which has been directly linked to how social and communicative competence of individuals with ASD is assessed by others^4^.

The relevance of prosodic processing to the quality of communication is not only due to the affective functions of prosody^5^, but also to its close interaction with syntactic analysis^6,7^. Several behavioral studies have looked at how prosodic features relevant for sentence parsing are processed in ASD^4,8–14^. In behavioral tasks involving prosodic phrasing, or chunking, individuals with ASD have been shown to either not differ from their neuro-typical peers^3,8,9,13,15^ or, on the contrary, to indeed have difficulties in prosodic processing^10,11^. The inconsistency of these findings may be partially attributed to a number of methodological challenges faced by behavioral studies on prosodic processing^2^. More sensitive experimental approaches, such as neuroimaging, could, therefore, shed light on whether prosodic processing involved in sentence parsing is in fact impaired in ASD.

To date, few neuroimaging studies of prosodic processing in ASD have been conducted. An early functional Magnetic Resonance Imaging (fMRI) study found that affective prosody failed to recruit right-hemisphere regions in an ASD group, suggesting that this group relies on a different cortical network for prosodic processing^16^. Other fMRI studies, however, have examined the prosodic skills of high-functioning autistic individuals and found that while nearly identical right-hemisphere regions were activated in ASD and typically developing populations during both affective and linguistic prosody tasks, additional brain areas, including the left supra-marginal gyrus were recruited in the ASD group^17,18^. The recruitment of these additional areas may reflect an overcompensation mechanism in which ASD individuals attend more closely to phonemic details^17^, perhaps due to less automaticity of language processing^18^. Another study, using near infrared spectroscopy, found that ASD subjects showed a similar right-hemisphere laterality of prosodic processing as neuro-typical subjects, despite demonstrating a prosodic impairment^19^. The authors theorized that the prosodic impairment may arise from the integration of acoustic features of language with semantic or syntactic factors. Together, these studies suggest that the neural substrates underlying affective and linguistic prosody in ASD are only partly overlapping with those in neuro-typical populations, and that the prosodic impairment may stem from integrating disparate features of language.

Whereas language impairment has been reliably observed in ASD, musical abilities in this group are consistently reported to be spared^20^ or even heightened^21–26^. Studies have also repeatedly provided evidence of overlapping, and possibly shared neurocognitive mechanisms underlying music and language perception in neurotypical populations^27^. Language and music processing rely heavily on both left- and right-hemisphere structures, including superior temporal, and inferior fronto-lateral regions in both hemispheres, although syntactic information is usually processed with a left-hemisphere weighting in language, and right-hemisphere weighting in music^28,29^. However, processing of prosodic features of language relies on right-hemisphere networks, similar to those responsible for tonal perception in music^30^. Thus, language, and specifically prosodic impairment in ASD in the absence of a corresponding music impairment, presents a curious dissociation.

Established theories about the nature of ASD neurocognition fail to explain this dissociation. The underconnectivity framework has been proposed based on several studies demonstrating a decrease in structural long-range, primarily frontal-posterior, but also interhemisphere, connections between brain regions in the ASD population^31–33^. The notion of long-range underconnectivity (and the resulting local over-connectivity) is in line with the behavioral findings of perceptual bias individuals with ASD have toward local, fine-grained characteristics of both auditory and visual input^34,35^. Models such as Weak Central Coherence^35^ (WCC) and Enhanced Perceptual Functioning (EPF)^36^ have been proposed based on this observation. The former suggests that the local bias in ASD occurs as a consequence of an impairment in processing global characteristics of input. The EPF, in contrast, emphasizes that the focus on local information reflects superior low-level perceptual processing that is independent of the presence or the absence of difficulties in high-level processing. Additionally, the theory asserts that higher-level features of perception do not interfere in tasks that can be processed locally for an ASD population^36^. However, WCC and EPF, as well as the corresponding proposal of long-range neural underconnectivity, fail to account for the observed discrepancy between language and music processing skills in ASD. Lai, Pantazatos, Schneider, and Hirsch^23^ came to a similar conclusion: they showed that in children with autism, the dissociation between spoken language and song processing was characterized by differences in left inferior frontal gyrus (LIFG) activation compared to their neurotypical peers, rather than being marked by deviated connectivity patterns. They suggested instead that the preserved musical abilities may be attributed to differences between the low-level auditory cues essential for music, on the one hand, and language, on the other, (consistent with more recent findings^37,38^), and that the language-specific low-level auditory information might not be received by the higher-level LIFG region due to an impairment in lower processing regions. Note, however, that Lai and colleagues^23^ studied a group of low-functioning children with autism (compared to an age-matched group of neurotypical children), some of whom were sedated, which presents a challenge for drawing conclusions about the nature of the dissociation between language and music in autism.

To address the questions arising from the inconsistent behavioral reports of prosodic impairments and conflicting explanatory models of ASD, and to specify the relationship between prosodic and music processing in this disorder, we used event-related potentials (ERP). ERPs provide high-resolution information about the timing, and about the amount of neural resources of cognitive processing (making this technique sometimes more sensitive to subtle processing differences compared to behavioral tasks). With respect to prosody, ERPs offer a well-established paradigm to study prosodic chunking, which, importantly, can be analogously used for the study of musical phrasing. Neurophysiological studies of prosodic phrasing revealed the so-called Closure Positive Shift (CPS) – a positive-going electrophysiological response elicited by intonational phrase (IPh) boundary perception in both spoken^39–43^ and written language^44,45^. IPhs are major prosodic chunks within a sentence, which normally correspond to syntactic phrases. IPh boundaries are marked by prosodic cues that may differ across languages and speakers, though in all cases, recognition of IPh boundaries plays an important role in the correct interpretation of a given sentence. The CPS is elicited immediately after the onset of the pause between two intonational phrases, can last for several hundred milliseconds, and typically has a distribution near the midline of the scalp^43^. Whereas the CPS component is thought to index prosodic chunking of preboundary phrase elements in real time, mismatches between prosodic chunks and syntactic phrasing requirements in so-called ‘garden-path’ sentences may elicit a subsequent bi-phasic N400/P600 response on disambiguating words^39^. This ERP pattern associated with processing of prosody-syntax mismatches provides an additional electrophysiological measure of successful prosody perception, independent of the CPS.

Several studies have also described a positive-going ERP waveform elicited close to phrase boundaries in music, which was said to resemble the CPS in language^46–49^. This so-called ‘music-CPS’, however, is characterized by a latency and duration different from that found for the language-CPS. More recently, the functional significance of this post-boundary music-CPS has been drawn into question, and an additional CPS-like response, named the boundary-onset music-CPS, has been observed during the musical phrase boundary for both musicians and non-musicians^50^. This response has a similar latency and distribution to the language-CPS, and is a prolonged positive shift. The similarity of these ERPs in language and music processing makes them useful tools for understanding differences between language and music cognition.

The present study is the first to investigate the CPS in response to both language and music in ASD. Importantly, the use of this experimental paradigm allowed us to make predictions disambiguating the different explanatory models proposed for the neurocognition in ASD in general and the dissociation between language and music skills in this group in particular. Based on existing research results, we have considered three possible outcomes. *(1)* The absence of differences in prosodic or musical phrasing between high-functioning adults with ASD and neurotypical individuals would be in line with the EPF model that does not imply an obligatory impairment in high-level cognitive processing mechanisms in ASD. *(2)* An impairment in both prosodic and musical phrasing reflected in the markedly different amplitude and/or latency of the two CPS components in ASD compared to those of the neurotypical group would be consistent with the general underconnectivity hypothesis and WCC. *(*3*)* Impaired prosodic phrasing along with intact musical phrasing would imply that the difference between prosodic and musical phrasing skills in ASD stems not from a domain-general chunking impairment, but rather from language-specific difficulties, perhaps stemming from differences in low-level acoustic characteristics between language and music^37^, such as the temporal complexity of the input^23^, or the integration of multiple levels of information in language^19^. Both options (*2*) and (*3*) would imply that, as a consequence of the predicted prosodic impairment, we would also see a deficit in processing prosodically cued garden-path structures in the ASD group, resulting in no differences between ERP responses to sentences with or without a prosody-syntax mismatch.

## Methods

### Participants

Thirty-three participants were tested as part of the study. Five participants’ datasets (two from the ASD group and three from the neurotypical group) were excluded from the analysis due to extensive EEG artifacts or due to being extreme outliers; the group descriptors below are representative only of the participants whose data were included in the analysis. Eleven participants (three women) had a diagnosis on the Autism Spectrum of High-Functioning Autism, Asperger’s Syndrome, or both. Diagnoses were made by physicians according to the Autism Diagnostic Observation Schedule (ADOS)^51^ and Autism Diagnostic Interview - Revised (ADI-R)^52^, or based on the criteria of ICD-10^53^ or the DSM-IV TR^54^. These participants were recruited from the Berlin/Brandenburg metropolitan area either through psychiatrist recommendation, or through advertisements in organizations related to autism. The ages of the ASD participants ranged from 23 to 54 with a mean age of 37.45 (SD 9.5).

Seventeen neuro-typical (NT) participants (seven women) were included in the analysis. All participants gave informed consent and were paid for their participation. Ethics approval was obtained from both the FU Berlin and Tulane University, and all methods were performed in accordance with the relevant guidelines and regulations. The neuro-typical participants ranged in age from 21 to 56 with a mean age of 28.47 (SD 8.25). Although the difference in ages of the two groups was significant (*t* = 2.571, *p* = 0.019), the results were virtually identical when analyzing an age-matched subset of neuro-typical behavioral and EEG data. Further, no age effects have been observed in the previous language-CPS studies, and while the absence of N400 and a more anterior P600 have been correlated with normal aging, those results were from a much older group (65–80 years)^55^. Thus, for purposes of statistical power, the larger group’s data will be reported. Exclusion criteria for both groups comprised history of neurological problems (aside from ASD for the ASD group), a family member with ASD (for the neuro-typical group), or a history of alcohol or drug dependence.

### Neuropsychological Battery

Participants were given a series of questionnaires, including the German translation of the Autism Questionnaire^56,57^, the Multiple Choice Vocabulary Test-B (MWB) test for Verbal IQ^58^, the Performance Testing System (LPS), Section 4 test for Non-Verbal IQ^59^, a Handedness Questionnaire^60^, and a brief in-house questionnaire assessing the musical background of participants.

The Autism-Spectrum Quotient (AQ) is a screening test designed to determine the degree to which an adult with normal intelligence has traits associated with the autistic spectrum, including assessments of social skills, attention to detail, and communication^56^. A cutoff of score of > 32 was used to classify those individuals with a high degree of ASD traits. None of the neuro-typical participants scored above 23. Section 4 of the LPS is a logical, non-verbal reasoning test. Only raw scores from this section are shown. The results of the neuro-psychological battery can be found in Table 1.

**Table 1:** Results of the neuropsychological battery by group: Verbal-IQ from the MW-B (Lehrl 1995), Non-Verbal from section 4 of the Leistungprüfsystem (Horn 1962); a German version of the Autism Quotient (Baron-Cohen *et al*. 2001); a Handedness Questionnaire adapted from Oldfield (1971). Results stated in: Mean (with standard deviation in parentheses), except for t and p values from the t-test.

	Group	Verbal-IQ	Non-Verbal LPS	AQ	Handedness
Mean (SD)	NT	108.06 (14.5)	31.1 (4)	13.88 (4.75)	79.14 (46.2)
	ASD	114.36 (13.6)	30.4 (6.9)	35.8 (9.37)	56.1 (64.97)
***t***	NT v ASD	1.167	0.287	7.183	1.022
***p***	NT v ASD	0.255	0.778	< 0.001***	0.322

### Stimuli

The experimental paradigm was developed based on the one used by Steinhauer and colleagues^39^ with language stimuli adapted from the study of Pannekamp and co-authors^61^ to make the task more appropriate for adult participants, and relied on the same stimuli used by Glushko and colleagues^50^. The sentences were grammatically all of the same form (# indicates the IPh boundary):

**Intransitive**: ‘Maxe bittet Tina zu lächeln, # und das Lied mitzusingen’.

*Maxe asks Tina to smile, # and to sing a song*.

**Transitive**: ‘Maxe bittet, # Tina zu grüssen, # und das Lied mitzusingen’.

*Maxe asks # to greet Tina, # and to sing a song*.

These sentences differed primarily in the argument structure of the second verb. In the above examples, the second verb in the Intransitive condition, *lächeln* (‘to smile’), is intransitive, while *grüssen* (‘to greet’), in the Transitive condition is transitive and takes ‘Tina’ as its accusative argument. The sentences follow the same word order, yet the intonation patterns with which these two sentences are spoken differ, aiding in the hearer’s syntactic parsing. Pauses at the IPhs were silenced, and edited for consistency to a duration of 600 ms.

In addition, we constructed prosodically and syntactically incongruous sentences (the ‘Mismatch’ condition) to test the validity of any prosodic phrase boundary processing impairment. If there are differences between the two groups’ language-CPS, we wanted to ensure that they were due to the impairment in processing phrase boundaries in language (shown by diminished garden-path effects), rather than more efficient processing of intonational phrase boundaries (shown by comparable garden-path effects). Thus, the Transitive and Intransitive sentences were carefully cross-spliced during the alveolar closure at the beginning of the affricate /ts/ in the word ‘zu’, so that the phrase up to that point came from the Transitive condition, while the phrase following that point was from the respective Intransitive sentence. This resulted in prosodically and syntactically incongruous sentences, such as:

**Mismatch**: *‘Maxe bittet, # Tina zu lächeln, # und das Lied mitzusingen’.

*Maxe asks, # to smile Tina, # and to sing a song.

In total, 48 sentences of each of the three conditions were produced, resulting in a total of 144 sentences.

**Figure 1:**
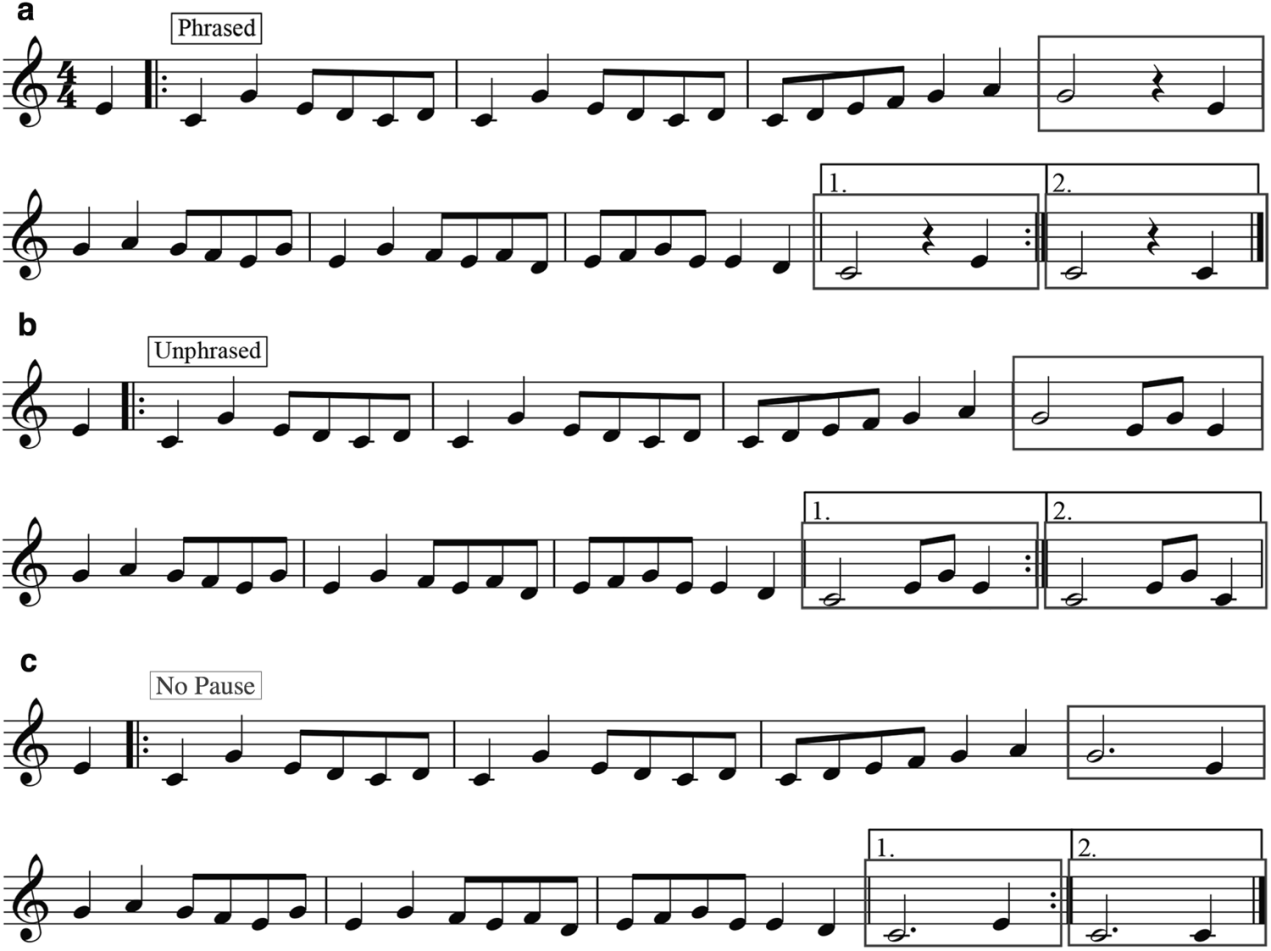
Sample notation of the music stimuli. Time periods of interest for the ERP analysis are highlighted with rectangles. Figure 1a: sample stimulus from the Phrased condition; Fig. 1b: Unphrased condition; Fig. 1c: No Pause condition.

The music stimuli were composed for this project by the first author, who is also a professional musician, and reviewed by a professional composer. The samples were composed in Sibelius First (Version 6.1.5, Avid Technology, Burlington, USA) and exported with a realistic acoustic piano sound. Each track included 16 bars at 100 beats per minute (bpm) with an upbeat. Each musical sample followed the same structure of two four-bar phrases creating an 8 bar piece, which was repeated. Three musical conditions were created for this study (Fig. 1):

**Phrased** (Fig. 1a): a pre-boundary half note followed by a pause (a quarter note rest lasting 600 ms)**;**

**Unphrased** (Fig. 1b): a pre-boundary half note followed by either a quarter note or two eighth notes (also 600 ms)**;**

**No Pause** (Fig. 1c): a boundary dotted half note of 1800 ms duration with gradual amplitude decay.

The names of the first two categories are used by convention, even though as Nan and colleagues^48^ acknowledge, the Unphrased condition is in fact ‘less phrased’, since other phrasing cues remain in addition to a pause or rest, including a lengthened phrase-final note, harmonic closure, and the structural convention of 4-bars. The third condition was designed to determine if a musical CPS associated with musical phrase boundaries is dependent on the presence of a pause (or rest). Further, previous studies had not examined the time window between phrases, which corresponds more closely to where the language-CPS occurs. However, in the absence of this third condition, the comparison of responses in this time window would be confounded by the presence of silence in one condition and additional notes in the other. No ‘Mismatch’ condition was used in the music part of the experiment due to the lack of a musical equivalent to the prosody/syntax mismatch. Importantly, the elicitation of the (language-)CPS is virtually independent of the presence (or absence) of violations or anomalies in the sentence^44^ and task requirements^39^. Therefore, whether a Mismatch condition was present in speech and not in music should have little to no impact on the elicitation of the CPS components.

### Procedure

All measurements were performed at the Dahlem Institute for the Neuroimaging of Emotion Laboratory at the Freie Universität Berlin. The stimuli were presented using Presentation® software (Neurobehavioral Systems, Berkeley, USA) in separate counterbalanced language and music blocks, with stimuli presented in randomized order. The entire procedure, including the completion of consent forms and questionnaires, lasted approximately 120 minutes. Following the presentation of each sentence, participants were asked “How natural did you find the last sentence?” Responses were provided on a five-point scale from “completely unnatural” to “completely natural”. This task mainly sought to determine if the ASD group was able to accurately perceive the violation in the Mismatch condition. The corresponding task for the music stimuli was to answer the question “How natural did you find the last piece of music?”, the main purpose of which was to maintain participant attention during stimulus presentation.

### EEG Recording and Analysis

All EEG recordings were conducted using 32 Ag/AgCl electrodes distributed according to the international 10–20 system using Brain Vision Recording Software (Brain Products GmbH, Gilching, Germany) at a sampling rate of 500 Hz. Electrodes were attached on both mastoids, of which the right mastoid was used as a reference electrode. Also, a ground was attached to the nape of the neck, and all impedances were kept below 10 kΩ.

EEG recordings were analyzed using EEProbe software (ANT Software BV, Enschede, Netherlands). Eye-blinks and facial movements were then corrected using a regression-based statistical algorithm, or rejected manually. Participants with more than 40% of trials rejected in any of the conditions (one participant with ASD and two neurotypical participants) were not included in the analysis. A band-pass FIR filter of 0.3–30 Hz (1001 points) was used to remove line noise and artifactual slow waves.

In the language stimuli, epoch measurements were time-locked to the beginnings of pauses marking phrase-boundaries. For CPS analyses in both language and music, multiple baseline correction regions were used to compare the stability of effects observed during different conditions (see Table 2). To quantify the CPS component, ERP responses were then measured across the 600 ms time windows (TWs) following the phrase boundaries. In the Mismatch condition, the prosody-syntax mismatch effects were assessed time-locked to the onset of the disambiguating second verb of the sentences. Intransitive and Mismatch conditions were contrasted using peak-to-peak analysis preceded by filtering of the EEG data with a 5 Hz low-pass filter. Local minima and maxima were identified for the time windows of 250 to 650 ms and 600 to 1200 ms, respectively, at electrodes Cz and Pz following the onset of the second verb. These electrodes were chosen due to the central and parietal distribution of the N400/P600 responses to garden-path sentences in our and previous studies^39,50^.

**Table 2:** Summary of the time windows (TW) and baseline corrections used for analysis of EEG data. All values expressed in milliseconds (ms).

Effect	Language		Music	
	CPS	N400/P600	Boundary-onset music-CPS	Post-boundary music-CPS
**Baseline** (**ms**)	−500 to 0; −50 to +50	None: peak-to-peak	−2000 to −1800; −1800 to −600	−2000 to −1800*
**TWs** (**ms**)	0 to +600	Min: +200 to +650 max: +600 to +1200	−450 to 0	+450 to +600

*A baseline-independent analysis was also used for this TW. See Supplementary Materials for details.

All epochs and baselines in the music experiment are stated in reference to the onset of the first post-boundary note (see Table 2). TWs for analysis included: the 450 ms preceding the onset of the post-boundary phrase-initial note for the boundary-onset music-CPS, and between 450 and 600 ms following the onset of this note (for the post-boundary music-CPS: see Supplementary Materials).

### Statistical Analysis

Statistical analysis of behavioral data was conducted using the statistical analysis software *R* (*R* Foundation for Statistical Computing, Vienna, Austria), and SPSS (Version 22, IBM, Armonk, USA). Behavioral responses were averaged by participant and condition, and then analyzed using two repeated measures 2 × 3 analyses of variance (ANOVA) with the two level between-subject factor Group (ASD vs. NT), and the three level within-subject factor Condition composed of the three language (Intransitive vs. Transitive vs. Mismatch) and music (Phrased vs. Unphrased vs. No Pause) conditions, respectively. Post-hoc comparisons between condition pairs for each group were performed with Two-Sample *t*-tests, using Bonferroni correction for multiple comparisons.

EEG data were grouped into regions of interest (ROIs) and averaged across electrodes for each condition and participant. Lateral ROIs included 18 electrodes, with 3 in each ROI: AntLeft: F3, F7, FC5; AntRight: F4, F8, FC6; CentLeft: C3, CP5, T7; CentRight: C4, CP6, T8; PostLeft: P3, P7, O1; and PostRight: P4, P8, O2. Midline comparisons were conducted using electrodes Fz, Cz, and Pz. Any participant whose average ERP response (lateral or midline) in any condition, language or music, fell more than +/−2.5 Standard Deviations away from the mean of the group was considered as an outlier and excluded from analysis. Then, repeated measures ANOVAs were conducted for all TWs for both lateral and midline ROIs. For the lateral electrodes, ANOVAs containing the factors Condition (language-CPS TW: Intransitive vs. Transitive; N400/P600 TW: Intransitive vs. Mismatch; Boundary-onset music-CPS TW: Phrased vs. No Pause), Hemisphere (L vs. R), AntPost (Ant vs. Cent vs. Post), as well as the between-subject factor of Group (ASD vs. NT) were conducted. For the Midline electrodes, similar repeated measures ANOVAs were conducted without the factor Hemisphere and using electrode site for the factor AntPost (Fz vs. Cz vs. Pz). Greenhouse-Geisser corrections for sphericity were applied where appropriate. Uncorrected degrees of freedom are shown accompanied by corrected *p* values. Effect sizes are reported for all main effects and interactions using Cohen’s *d*.

Due to the widely recognized heterogeneity of ASD, and the relatively small sample size, each EEG data set was averaged across lateral and midline electrodes, respectively, and then tested for normal distribution using Kolmogorov-Smirnov distribution tests. This resulted in normal distributions for all analyses with the exception of the Midline analysis for the boundary-onset music-CPS data subset. Therefore, for this analysis, non-parametric Wilcoxon Signed Rank and Mann-Whitney U tests were used instead of ANOVA.

## Results

### Behavioral Results

**Figure 2:**
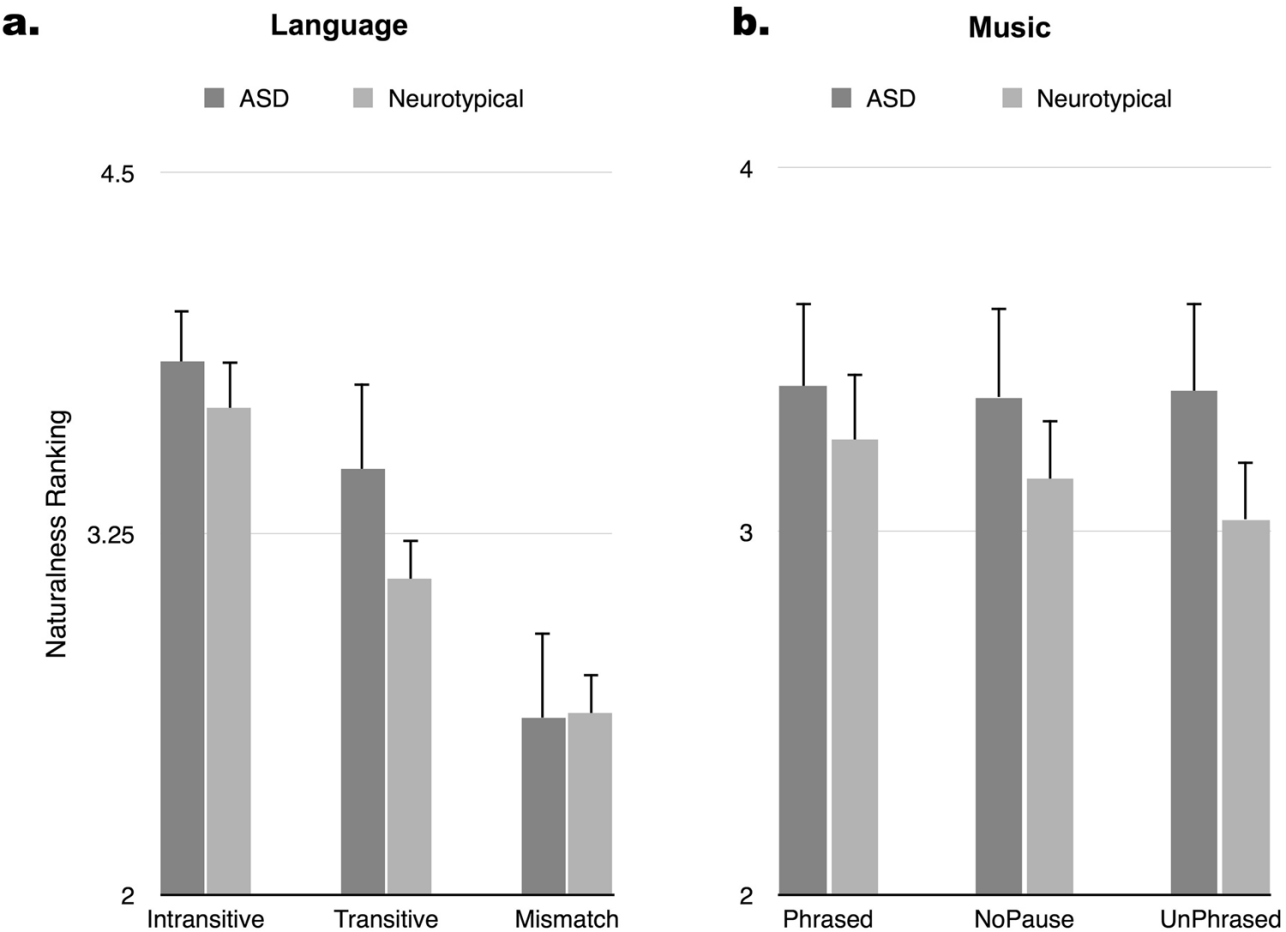
Results of the behavioral experiment. Figure 2a: mean naturalness ratings (1 – least natural, 5 – most natural) with standard error bars of the sentences in the language part of the experiment by group. There were no between-group differences in naturalness ratings, but both groups rated the Mismatch condition as the least natural and participants also rated the Transitive condition as less natural than the Intransitive condition. Figure 2b: responses to the question “how natural did you find the last piece of music?” (1 – least natural, 5 – most natural). There were no differences observed by condition or group.

For the language responses (Fig. 2a), the Intransitive condition was ranked as most natural (Mean: 3.749, SD: 0.66), while the Transitive sentences (Mean: 3.244, SD: 0.67) were ranked as less natural than the Intransitive ones. The Mismatch condition, which contained the violation, was ranked least natural of the three (Mean: 2.622, SD: 0.79). Main effects of Condition were significant at *p* < 0.001 for the global ANOVA (*F*(2, 25) = 32.874, *p < *0.001) and for all *t-*tests following Bonferroni correction (Intransitive vs. Transitive: *t*(27) = 6.121, *d* = 0.763; Intransitive vs. Mismatch: *t*(27) = 6.536, *d* = 1.55; Transitive vs. Mismatch: *t*(27) = 4.283, *d* = 0.852). No significant main effects of Group were observed, nor was an interaction of Condition × Group. The lack of between-group differences was not entirely unexpected, given that previous studies have reported inconsistent results of behavioral experiments on prosody (one of the motivations for the use of ERPs).

For the ratings of the music stimuli (Fig. 2b), no main effects nor interactions of Group nor Condition were observed, however, none of the musical stimuli contained violations, and only differed in the degree of phrasing. Because the purpose of the behavioral task in the music conditions was to maintain the attention of the participants, these results are consistent with our expectations. Overall, no between-group differences were observed when behavioral measures were used.

### EEG Results: Language

**Figure 3:**
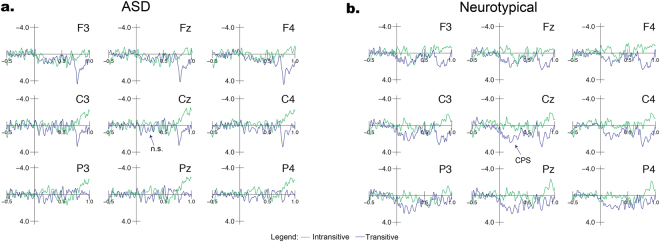
Brain-electric responses (group averages) to the Intransitive (without IPh boundary) and Transitive (with IPh boundary) conditions using a baseline of −500 to 0 ms. Figure 3a shows ASD group responses, while Fig. 3b shows neurotypical group responses. A clear CPS was observed in the Neurotypical group in response to the Transitive condition, while differences between conditions were not significant for the ASD group.

**Figure 4:**
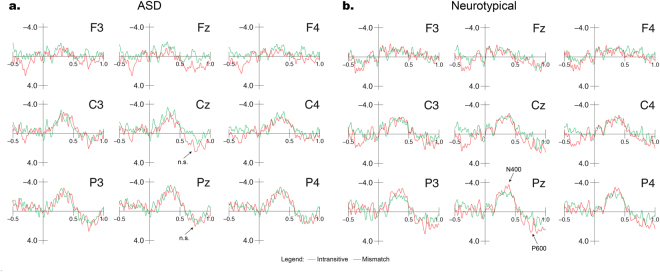
Brain-electric responses (group averages) to the Intransitive and Mismatch comparisons during the TW of expected Garden Path effects. Baseline correction of −50 to + 50 ms was used for demonstration purposes, while peak-to-peak comparisons were used for analysis. Figure 4a shows ASD group responses which did not differ by Condition. Figure 4b shows neurotypical group responses which contain Garden Path (N400/P600) effects.

The EEG results of the language task are summarized in Figs 3 and 4. For the sake of brevity, only the results of the −500 to 0 baseline correction for the first CPS are reported, except when meaningful differences were seen between baseline corrections.

### Language-CPS

As can be seen from the EEG waveforms in Fig. 3b, a clear language-CPS was observed in the NT group in response to the Transitive condition. This positivity started at the onset of the boundary pause and had a broad centro-parietal scalp distribution. The clear positivity for the NT group contrasted with the less distinct positivity with a smaller amplitude and more limited distribution for the ASD group (Fig. 3a).

A significant main effect of condition was observed for ANOVAs of both lateral (*F*(1, 26) = 11.619, *p* = 0.002, *d* = 0.883) and midline (*F*(1, 26) = 7.807, *p* = 0.01, *d* = 0.771) electrodes between the Intransitive and Transitive conditions, confirming the interpretation based on visual inspection of the language-CPS. The Condition × Hemisphere interaction (*F*(1, 26) = 3.648, *p* = 0.067, *d* = 0.197) approached significance, suggesting that the language-CPS responses were slightly larger over the right-hemisphere. Moreover, a Condition × Group interaction was found for comparisons at both lateral (*F*(1, 26) = 5.163, *p* = 0.032, *d* = 0.515) and midline (*F*(1, 26) = 5.192, *p* = 0.031, *d* = 0.535) sites. Post-hoc analysis conducted for each group showed that for the NT group, there was a highly significant effect of Condition with a large effect size (lateral: *t*(16) = 5.138, *p* < 0.001, *d* = 1.388; midline: *t*(16) = 4.567, *p* < 0.001, *d* = 1.34), while for the ASD group, the effect of Condition was not significant and the effect size was small (lateral: *t*(10) = 0.626, *p* = 0.545, *d* = 0.259; midline: *t*(10) = 0.285, *p* = 0.781, *d* = 0.118). That is, despite the apparent presence of a slight positive response to the Intransitive condition for the ASD group, this effect was not significant. Additionally, we performed simple linear regressions to investigate whether age and VIQ scores of participants in each of the experimental groups could predict the variability of the language-CPS amplitude at midline electrodes, but none of the results was statistically significant.

### Garden-Path Effects

Figure 4 shows the grand average waveforms for the ASD (Fig. 4a) and NT (Fig. 4b) groups for the Intransitive and the Mismatch conditions, which are acoustically identical during this period. The baseline of −50 to +50 ms relative to the onset of the verb is used for demonstration purposes (uncorrected peak-to-peak comparisons were used for statistical analysis). While no effects were significant at electrode Cz, a main effect of Condition (Intransitive vs. Mismatch) approached significance at electrode Pz (*F*(1, 26) = 4.193, *p* = 0.051, *d* = 0.466), as did the interaction of Condition × Group at the same electrode (*F*(1, 26) = 3.591, *p* = 0.069, *d* = 0.301). Because a detailed comparison of the violation responses was crucial to examining ASD prosodic processing, follow-up pairwise *t-*tests were conducted to determine if garden-path responses were present for both groups. These pairwise *t-*tests conducted for the two conditions showed that for the NT group, there was a significant effect of Condition with a large effect size (*t*(16) = 2.727, *p* = 0.015, *d* = 0.81) due to an N400/P600 response to the Mismatch condition, while for the ASD group, the difference in responses to the two conditions was not significant and the effect size was negligible (*t*(10) = 0.143, *p* = 0.889, *d* = 0.026). We also performed simple linear regressions to investigate correlations between garden-path effects at Pz for both groups with age or VIQ score, but none of the results reached statistical significance.

### EEG Results: Music

All TWs and baselines are stated in milliseconds relative to the onset of the first note following the phrase boundary in the Phrased and No Pause conditions, and the corresponding note in the Unphrased condition.

### Boundary-Onset Music-CPS

The results of the two baseline corrections were nearly identical; therefore, we report the results using a −1800 to −600 ms baseline, mentioning the results of the −2000 to −1800 baseline only in cases of meaningful differences between the two analyses. Due to the presence of confounding notes ‘filling in’ the phrase boundary in the Unphrased condition, the analysis of this TW focused on comparisons of the Phrased and No Pause conditions.

**Figure 5:**
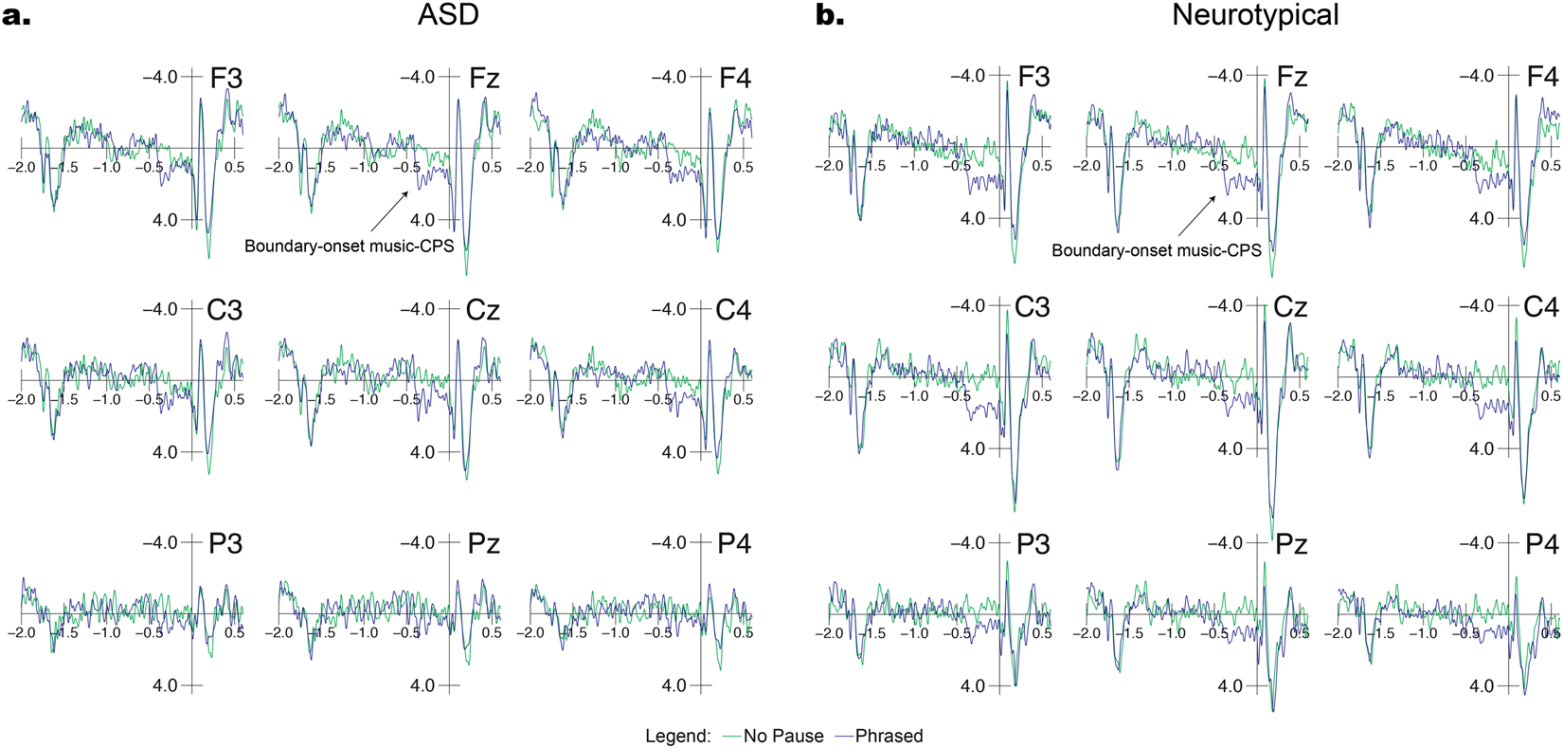
Brain-electric responses (group averages) to the boundary-onset music-CPS using a baseline of −1800 to −600 ms relative to the onset of the post-boundary phrase. Figure 5a shows ASD group responses, while Fig. 5b shows Neurotypical group responses. Both groups show a clear boundary-onset music-CPS in response to the Phrased condition.

As can be seen from the comparison of these two conditions in Fig. 5, both groups showed a positive shift during the phrase boundary in response to the Phrased condition. This shift resembled the language-CPS, as it was a prolonged positivity occurring during the pause between two phrases, with a central maximum. Overall, however, this response had a slightly more anterior distribution than the language-CPS in the present study (at the same time, note that a fronto-centrally distributed language-CPS has previously been reported by several studies^42,43,61,62^).

In the TW of −450 to 0, the two condition (Phrased vs. No Pause) ANOVA showed significant main effects of Condition for both lateral (*F*(1, 26) = 15.739, *p* = 0.001, *d* = 0.821), and midline comparisons (Wilcoxon Signed Rank *p* = 0.001, *d* = 0.933). This effect appeared smaller in the ASD group through visual inspection of the waveforms, yet, importantly, there was no significant Condition × Group interaction at lateral sites (*F*(1, 26) = 1.615, *p* = 0.215, *d* = 0.246) and there were no significant differences between groups for either condition at midline sites (Mann Whitney U Phrased: *p* = 0.306, *d* = 0.416; Mann Whitney U No Pause: *p* = 0.578, *d* = 0.065). There were also significant main effects of Condition for the NT group (Wilcoxon Signed Rank NT *p* = 0.001) and the ASD group approached significance (Wilcoxon Signed Rank ASD *p* = 0.05) Additionally, there was a significant Condition × AntPost interaction at lateral sites (*F*(1, 26) = 5.864, *p* = 0.01, *d* = 0.75), confirming the more anterior distribution of effects.

## Discussion

### Behavioral Results

We found no behavioral differences between groups in either the music or language tasks. Both the NT and ASD groups rated the (language) Mismatch condition as the least natural. The absence of group differences was somewhat contrary to our expectations: reconciling prosodic and syntactic information from disparate processing pathways in ASD is counter to predictions based on underconnectivity, and Weak Central Coherence (WCC). However, both groups also rated the Transitive condition as less natural than the Intransitive condition. A possible reason for this could have been the silence during the pause at the first IPh of these sentences. The consistent, relatively long (600 ms) duration of the pause at the first IPh in the Transitive and Mismatch conditions may have influenced naturalness rankings by making the sentences appear edited. If so, the violation present in the Mismatch condition may not have been the only factor causing the groups to rank it as least natural. However, many sentences prior to editing already contained pauses of approximately the same duration, and other language-CPS studies^61^ have used stimuli with comparable IPh pause lengths. As mentioned earlier, behavioral measures of prosody in ASD have resulted in inconsistent findings^2^. Further, behavioral rankings may reflect a variety of cognitive mechanisms, which was one of the motivating factors in our decision to use the more sensitive ERP technique to study prosody in an ASD group.

### Language-CPS and Garden-Path Effects

A language-CPS was clearly present for the NT group in response to the Transitive condition, which contained an IPh boundary (as compared with the Intransitive condition, which did not). In line with previous studies^39,43^, the language-CPS was elicited almost immediately following the onset of the pause, and was characterized by a broad centro-parietally maximal distribution near midline electrodes, lasting between 300 and 500 ms, which was somewhat larger over the right hemisphere. These findings are consistent with those of previous language-CPS results in distribution, duration, and amplitude^39,43^. There was also a significant interaction of Condition × Group and follow up tests showed no differences in responses to the different conditions for the ASD group. While the ASD group appeared to show a slight CPS in response to the Transitive condition, this response was not significant. The lack of a language-CPS response for this group shows that prosodic chunking ability is impaired in individuals with ASD, consistent with the perceptual prosodic challenges often noted by individuals with these disorders^2,3^. While previous behavioral results have been inconsistent in describing the prosodic phrasing impairment in ASD, the use of more sensitive ERP measures in the present study confirms the presence of a linguistic prosody impairment for this group. The present study is the first to measure CPS data in ASD individuals, and to provide electrophysiological evidence of a prosody-based chunking impairment in this group.

Significant bi-phasic N400/P600 responses for the neuro-typical group were identified following the incongruous syntactic/prosodic splicing in the Mismatch condition, again replicating previous findings of similar experiments^39^. By contrast, no garden-path ERP effects were observed in the ASD group. The absence of violation responses in this group suggests that the differences between the Mismatch rankings and those of the other conditions in the behavioral results may have been partially due to factors other than recognition of the prosody-syntax mismatch, such as the presence of an artificially silenced pause with a consistent duration. The inconsistency of the behavioral measures with the ERP data confirms one of our motivations for the study: that more sensitive electrophysiological measures can provide new insights in the investigation of prosody in ASD. Further, the absence of garden-path responses in this group is in line with previous research reporting N400 and P600 differences in ASD. Braeutigam and colleagues^63^, summarizing previous research, found that ASD N400 responses vary substantially by task, with some studies finding no N400 effect, and others finding delayed effects. Recent studies have also failed to find a P600 response unless ASD participants were expressly told to focus on the degree of semantic implausibility in the sentences presented^64,65^. These previous studies demonstrate broader patterns of language pathology in ASD; in the present study, the absence of N400/P600 responses can have a twofold interpretation. First of all, given the non-significant language-CPS response to transitive compared to intransitive sentences in participants with ASD, the absence of a typical N400/P600 pattern in sentences with a syntax-prosody mismatch is not surprising and primarily reflects participants’ failure to process prosodic boundaries (i.e., leading to their inability to detect the associated syntax-prosody mismatch). In addition to this, it would be plausible, based on the underconnectivity hypothesis, to assume that the prosodic chunking impairment is accompanied by a distinct inability to integrate prosodic and syntactic information within a sentence. However, whether such an integration problem is present in ASD remains an open question.

### Boundary-Onset Music-CPS

The present study is the first to identify a positive shift lasting several hundred milliseconds during musical phrase-boundaries for individuals with ASD. This response did not statistically differ between groups. While the distribution of this boundary-onset music-CPS was more anterior than the language-CPS, its time-course was more similar to the language-CPS than that of the previously described post-boundary music-CPS, which we and several previous studies failed to find^66,67^ (for reasons why the interpretation of a post-boundary music-CPS may be problematic, see Supplementary Materials and Glushko *et al*.^50^; see also the absence of such an effect in the group of non-musicians in the study by Neuhaus and colleagues^43^). The boundary-onset music-CPS reported here and elsewhere^50,68^ occurs during the phrase boundary, as opposed to 500-600 ms following the first post-boundary note. Additionally, the fact that it is a relatively slow positive shift, as opposed to being a single peak-like positivity, suggests that this component is the musical equivalent to the language-CPS, which in some previous studies showed a comparable frontal distribution^42,61,69^. Together, these two responses reflect a domain-general neurocognitive mechanism of chunking^50^.

In both language and music, the acoustic cues signaling phrase boundaries have similar characteristics. The phrase final note or syllable tends to be lengthened, accompanied by a change in pitch, and followed by a pause. In music, there are the additional features of strong beats, as well as other components of metrical hierarchies^70^, that influence probable locations of phrase boundary divisions. The observed boundary-onset music-CPS likely represents the chunking of melodic or rhythmic phrases based on these cues, similar to the language-CPS. The fact that the positive shift was only observed in the condition with a musical pause, as opposed to the condition in which the phrase final note was held, suggests that the pause is crucial for the chunking of musical phrases. Whether the boundary-onset music-CPS can be elicited in the absence of a musical pause (and, if so, under which conditions) remains unresolved. Note, however, that in language, where the CPS closely follows the morphology of the boundary-onset music-CPS, the response is produced at phrase boundaries even in the absence of a pause, provided that other phrase final characteristics are present^39^.

### Dissociation between music and language processing in ASD

In the present study, we reported impaired processing of prosodic phrase boundaries along with preserved processing of musical phrase boundaries in ASD. Despite the CPS components, observed in both language (language-CPS) and in music (boundary-onset music-CPS), reflecting a domain-general neurocognitive mechanism of chunking^50^, the discrepancy between the elicitation of these ERPs in ASD presents a compelling dissociation.

The dissociation in the elicitation of the CPS in these two domains could plausibly stem from the inability of the higher-level chunking mechanism to access and rely on the lower-level perception of phrase boundary cues. In music, fine-grained pitch discrimination is crucial for the perception of harmony and consonance^37^. The ability of individuals with ASD to process fine-grained, local information, such as pitch and note changes within a melody (for a review, see^35^), would also allow for effortless pause perception at musical phrase boundaries. Therefore, music-specific phrase boundary processing (as reflected by the boundary-onset music-CPS) would be unimpaired.

In contrast to music, prosodic input presents a known processing difficulty for individuals with ASD^1^ and calls for increased activation of areas responsible for perception of low-level features of language (compared to the neurotypical population)^18^. While music processing as a whole relies on fairly exact perception of pitch changes, for language sentence prosody processing, coarser pitch change perception is sufficient^37^ and, in fact, might be essential for adequate speech analysis. The over-focus on fine-grained input features observed in individuals with ASD^34,35^ might actually be disadvantageous for their language processing abilities, driving attention away from prosody towards more rapid changes in phonemic information^17^ (which are not typical for music, see also^38^). Language perception relies on coordinating distinct slow (i.e. prosodic) and more fine-grained (i.e. phonemic) levels for successful parsing^38^, suggesting that an impairment in linking the two levels could also be responsible for the observed prosodic impairment. This interpretation of the dissociation between prosodic and musical phrasing in ASD is in line with the EPF model, which sees the local bias as a primary (rather than as secondary to the weak coherence) characteristic of ASD^36^. It also potentially fits the idea of local neural over-connectivity in ASD^31–33^ that would imply a right-hemispheric local over-connectivity allowing for proper harmony^28^ and fine-grained pitch^71,72^ processing as well as left-hemispheric local over-connectivity producing the bias towards phonemic analysis^17^. While it seems that the presence of the boundary-onset music-CPS in ASD would not easily fit predictions of underconnectivity, if the impairment caused by underconnectivity is one of integrating low-level and higher-level information together, this could explain the observed dissociation between language and music abilities in ASD. The possibility that superior pitch discrimination skills serve as an overcompensation mechanism boosting initially deficient phrasing abilities of individuals with ASD in the music domain, while the same overcompensation would not be successful in the language domain due to the relative independence of phonemic and prosodic levels, remains open and requires further investigation. It is clear, however, that in the language domain, individuals with ASD experience difficulties in prosodic chunking and these difficulties negatively affect their language comprehension skills (as reflected in the absent response to syntax-prosody mismatches in our study). This result is in line with previous behavioral reports of impaired prosodic chunking and descriptions of general linguistic abilities in ASD^2,10^.

## Conclusions

The present study is the first to investigate the electrophysiological responses to prosodic and music phrase-boundaries in individuals with autism spectrum disorders (ASD). While previous behavioral studies of ASD prosodic abilities have been contradictory and inconclusive, the use of the ERP technique strongly suggests a prosodic processing impairment in this group, with no corresponding impairment in music. In fact, the presence of nearly identical responses to music from the two groups provides empirical evidence that musical phrase structuring ability is preserved in ASD. This is also the first study to show a positive shift during the musical phrase boundary in individuals with ASD, similar to that found in neuro-typical individuals. This positivity may serve a similar function to the language-CPS, indexing a chunking mechanism that aids hierarchical and metrical structuring of musical input into cohesive phrases, while making predictions about what will come next. This interpretation implies that the dissociation between music and language processing abilities, particularly phrase boundary recognition, in ASD, stems from the differences between the acoustic properties of language and music and is potentially due to the allocation of neurocognitive resources to features of language other than prosody.

Both language and music abilities rely on the integration of overlapping networks incorporating various left and right hemisphere structures responsible for acoustic perception and rule-based operations, yet in ASD language ability is impaired, while music ability is not. Further research is needed to address the interaction of low-level and hierarchical features of music, as well as therapeutic avenues for reducing prosodic impairment, including whether musical training plays a role in improving prosodic processing and production, and if strategies for identifying prosodic phrase boundaries can decrease prosodic ambiguities in ASD.

## Electronic supplementary material

Supplementary Materials
