# Supplementary material for: Language and music phrase boundary processing in Autism Spectrum Disorder: An ERP study
Source: Sci Rep. 2017 Oct 31;7:14465. doi: 10.1038/s41598-017-14538-y (PMC5663964; doi:10.1038/s41598-017-14538-y)
Supplement: Supplementary file 1 — Supplementary Materials [file 41598_2017_14538_MOESM1_ESM.pdf]

Language and music phrase boundary processing in Autism Spectrum Disorder: An ERP study (Supplementary Materials)

John DePriest[1,2]*, Anastasia Glushko[1,4], Karsten Steinhauer[3,4], Stefan Koelsch[1,5]

[1]Freie Universität Berlin, Berlin, Germany

[2]Program in Linguistics, Tulane University, New Orleans, Louisiana, United States of America

[3]School of Communication Sciences and Disorders, McGill University, Montreal, Quebec, Canada

[4]The Centre for Research on Brain, Language and Music (CRBLM), Montreal, Quebec, Canada

[5]University of Bergen, Bergen, Norway

[*] Corresponding author

Full address: Tulane University, Human Language & Technology Lab, Newcomb Hall #401, New Orleans, LA 70118

Telephone: 504-862-3046

E-mail: jdepries@tulane.edu (JD)

Methods

Due to the relatively small sample size of the ASD group, we investigated individual variability within language-CPS responses to determine whether the observed results were driven by the established heterogeneity of the disorder. Also due to the relatively small size of the ASD group, and to further determine whether the lack of language effects in the ASD group were due to sample size or group differences, we conducted additional analyses using a smaller sample of the NT group ($N = 11$), which consisted of *t*-tests of EEG responses to the language-CPS time window.

Due to issues with baseline correction for the post-boundary music-CPS TW, an additional baseline independent analysis was conducted by subtracting averages from the post-boundary epoch from the TW immediately preceding it (330 to 450 ms). For the lateral electrodes, ANOVAs containing the factors Condition (Post-boundary music-CPS: Phrased vs. No Pause vs. Unphrased), hemisphere (L vs. R), AntPost (Ant vs. Cent vs. Post), as well as the between-subject factor of Group (ASD vs. NT) were conducted.

Results

Language-CPS

For the smaller neurotypical sample investigation of the language-CPS, we found that the smaller NT group ($N=11$) had a significant main effect of Condition at lateral ($t(10) = 5.907$, $p < 0.001$), and midline sites ($t(10) = 4.826$, $p = 0.001$). We can contrast this with the results of the t-tests for the same condition for the ASD group: lateral: $t(10) = 0.626$, $p = 0.545$; midline: $t(10) = 0.285$, $p = 0.781$. As we see even from a smaller NT group, the difference between conditions is highly significant, while that of the ASD group does not even approach significance.

Post-boundary Music-CPS

The grand average waveforms of all three conditions in the post-boundary music-CPS TW using a baseline from -2000 to -1800 ms are shown in Figure 5. No positivity was observed in response to either the Phrased or the No Pause condition where the post-boundary music-CPS has previously been described. The lack of relative

positivity in the Phrased condition may be due to the placement of the baseline over two seconds prior to the analyzed epoch. Thus, in order to determine whether this was the issue, a baseline independent analysis was also conducted by averaging each condition and electrode for each participant. Then, matching electrode and condition averages from the TW of +330 to +450 ms were subtracted to mimic baseline correction.

For the global ANOVAs of the baseline independent analysis, there was a significant main effect of Condition at lateral ($F(2, 56) = 3.577$, $p = 0.048$), but not midline ($F(2, 56) = 1.229$, $p = 0.289$) sites. The results of the pairwise comparison between the Unphrased and Phrased as well as between No Pause and Phrased conditions, however, did not result in any significant main effects. Only the comparison of Unphrased and No Pause showed a significant main effect of Condition similar to that from the global ANOVA (lateral: $F(1, 28) = 4.608$, $p = 0.037$, $d = 0.655$; midline: $F(1, 28) = 1.331$, $p = 0.258$, $d = 0.344$), showing that the global effects were largely driven by the comparison of the No Pause and Unphrased conditions. This effect was due to a greater difference between the TW of analysis and the subtracted TW for the Unphrased condition (which was slightly more positive in the post-boundary music-CPS TW) than the No Pause condition (which was slightly more negative in the same TW).

Discussion

Post-boundary Music-CPS

In the present study, no post-boundary music-CPS was observed for either group for any of the conditions, with either baseline corrected, or baseline-independent analyses. In fact, while there was a statistical difference between conditions using the baseline independent analysis, it was due to a larger difference between the TW of analysis and the TW preceding it in the Unphrased condition, as opposed to the Phrased condition, as would be predicted for this effect. In the absence of any CPS-like responses in the post-boundary time window, and given that no differences were observed with the baseline corrected analysis, the status of the post-boundary music-CPS must be drawn into question.

As proposed by Glushko and colleagues (2016), the previously described post-boundary music-CPS is

likely due to auditory evoked potentials (P200) of greater amplitude in the Phrased condition. Indeed, in the present study, the Unphrased condition shows reduced P2 effects following the phrase boundary in comparison to other conditions. While Neuhaus et al. (2006) address this possibility, they only mention that it is unlikely that the post-boundary music-CPS reflects the *first* P200 following the pause. This and other previous studies (see Glushko et al. 2016 for a complete reference list) did not have consistent note lengths in the stimuli, allowing for the possibility that the positivity could be due to sequential notes. Moreover, several studies have also failed to find it in expected environments (e.g. Istók et al. 2013; Hoshi-Shiba et al. 2014; as well as the absence of the effect in the group of non-musicians in Neuhaus et al. 2006). The low robustness of this ERP component suggests that its presence may be due to a potential confound, or that it is a phenomenon extremely sensitive to musical environment.

The absence of a post-boundary music-CPS in either group in the present study suggests that this effect may be either extremely sensitive to musical context, or due to confounding features of stimuli. Several studies have failed to observe this effect in predicted environments, and despite being a posterior positivity, neither the latency nor the duration of the component resemble the CPS observed in language, suggesting that this effect may index other cognitive processes.

Supplementary Materials Figure 1

Brain-electric responses (group averages) to the post-boundary music-CPS using a baseline of -2000 to -1800 ms relative to the onset of the post-boundary phrase. Neither group showed a post-boundary music-CPS in response to the either the Phrased condition or the No Pause condition.

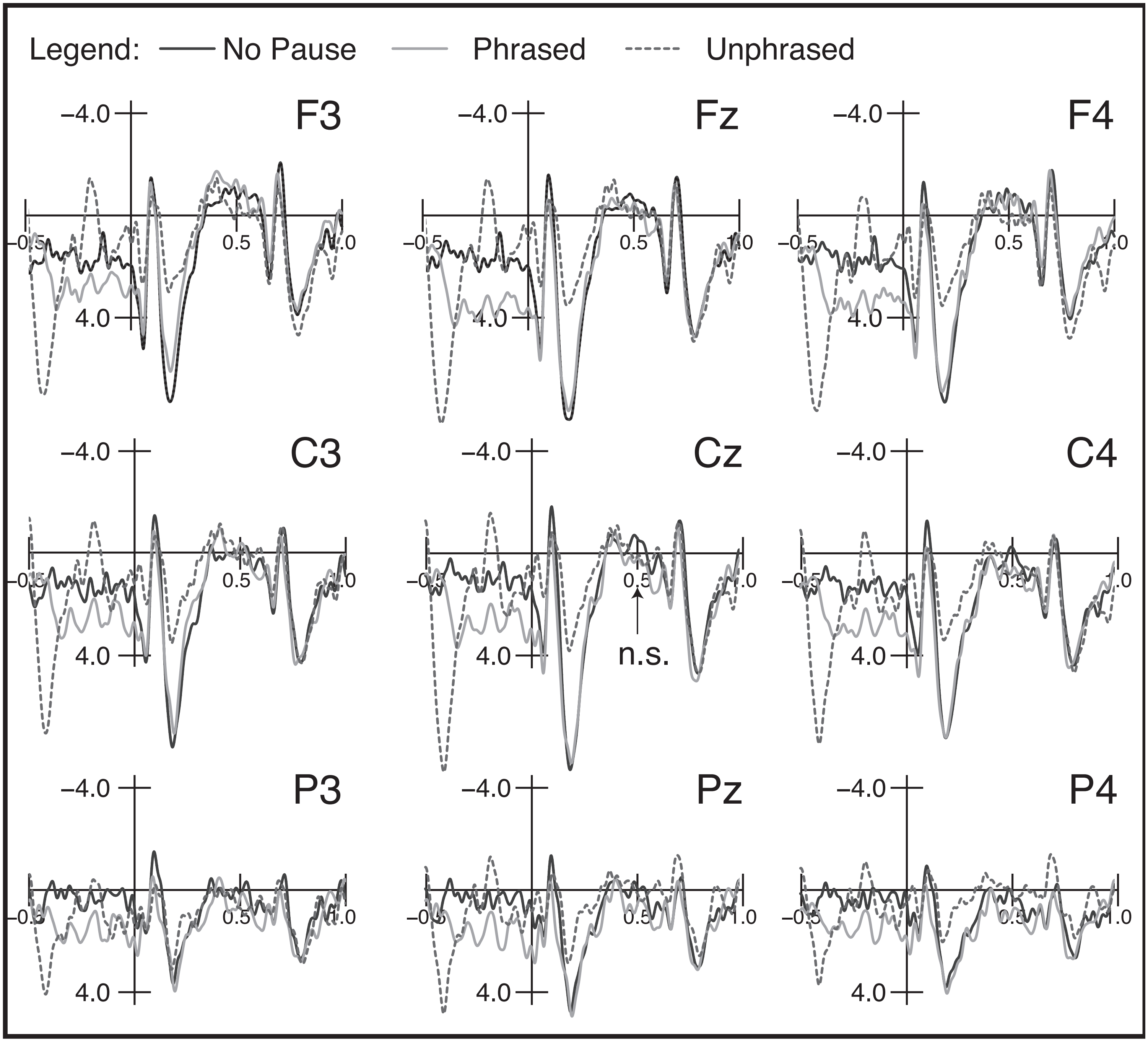

Legend:
No Pause
Phrased
Unphrased
F3
Fz
F4
C3
Cz
C4
P3
Pz
P4
−4.0
4.0
−0.5
0.5
1.0
n.s.

Supplementary Materials Figure 2

Brain-electric responses with Standard Error for both neuro-typical (A) and ASD (B) groups for both Transitive and Intransitive conditions.

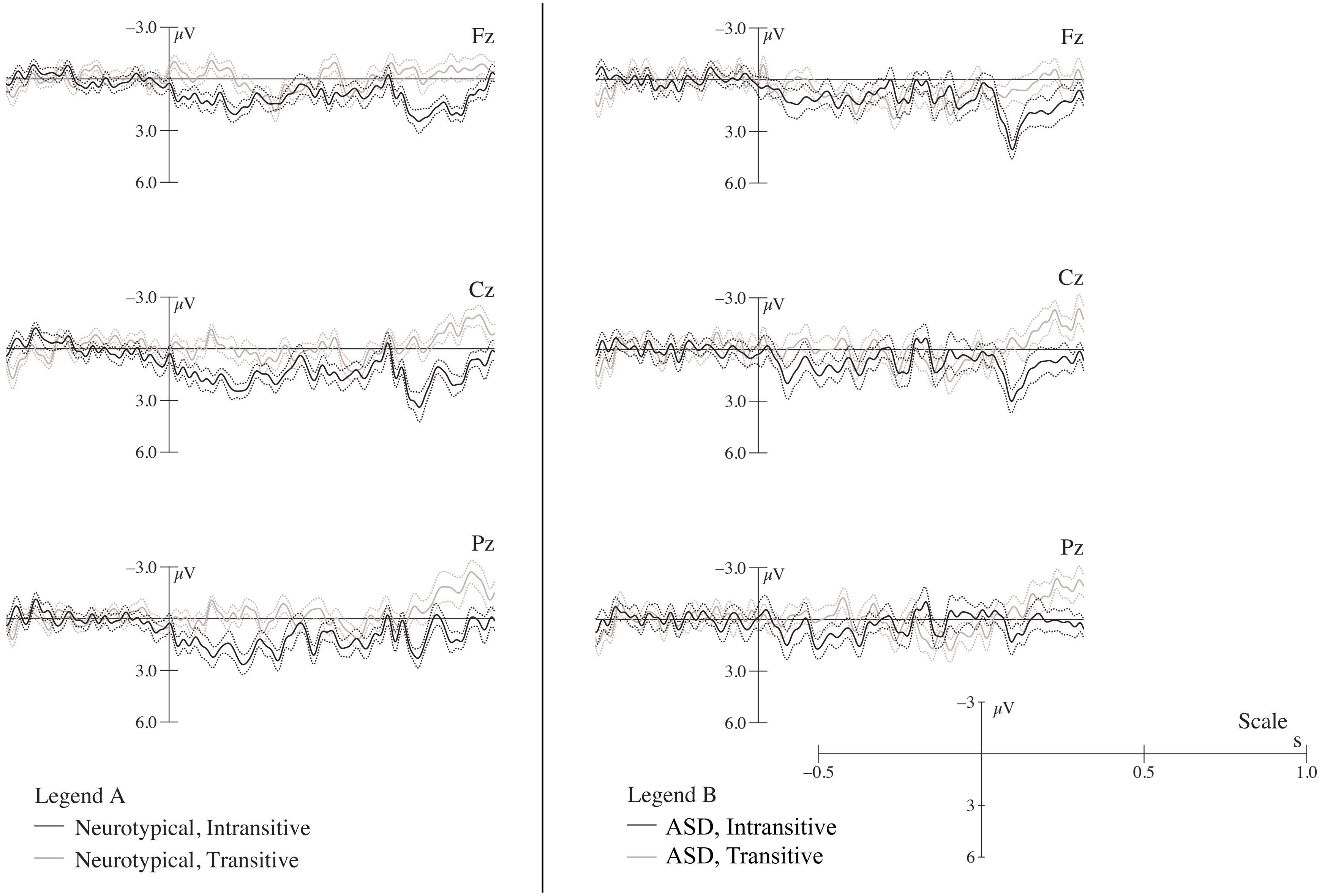

Fz
Cz
Pz
−3.0
3.0
6.0
μV
Fz
Cz
Pz
Scale
s
−3
3
6
−0.5
0.5
1.0
Legend A
Neurotypical, Intransitive
Neurotypical, Transitive
Legend B
ASD, Intransitive
ASD, Transitive
